# Exploring the paradigm of depressive disorders through an evolutionary and biopsychosocial lens

**DOI:** 10.1192/j.eurpsy.2023.1855

**Published:** 2023-07-19

**Authors:** T. Ochi, A. J. Loonen, G. G. Simutkin, N. A. Bokhan, A. N. Kornetov, S. A. Ivanova

**Affiliations:** 1 PharmacoTherapy, -Epidemiology & -Economics, University of Groningen, Groningen, Netherlands; 2 Mental Health Research Institute, Tomsk National Research Center; 3 Fundamental Psychology and Behavioral Medicine, Siberian State Medical University, Tomsk, Russian Federation

## Abstract

**Introduction:**

Depression can be considered to be a common psychological response to adversity or loss from which an individual may recover quickly based on a natural resilience mechanism. In major depressive disorder, however, we see that biopsychosocial factors exist that can prevent this natural resilience mechanism from taking effect.

**Objectives:**

To investigate neurotransmitter pathways linked with antidepressant response, genetic epidemiological studies and a literature assessment of biopsychosocial factors were conducted.

**Methods:**

Newly admitted patients with a depressive episode according to the criteria of ICD-10 (F32 or F33) who had not been on antidepressant medication for at least 6 months were recruited. More than half the patients have never been treated with antidepressant medication during their entire life. The patients’ depression was of at least moderate severity as measured by the Hamilton’s Depression Rating Scale (HAMD-17).

To determine the effect of adrenergic pathway genes to antidepressant response, the outcome was measured by the difference in HAMD-17 score between entry and two weeks of treatment after two and four weeks of treatment and entry and four weeks of treatment. Multiple linear regression was conducted to identify the independent factor associated with ΔHAMD-17 between the three time periods, including age, sex, depression diagnosis, type of antidepressant taken and selected SNPs.

Literature assesement utilised a snowball technique, building on prior literature reviews conducted. The selection of included literature was determined by the authors.

**Results:**

The Tomosk cohort was mainly women, with less than 20% of patient being male. The cohort was dynamic thus the number of participants involved in each investigation varied. Most patients took SSRIs, specifically sertraline, paroxetine, escitalopram, fluoxetine and fluvoxamine. Comparing the medication taken, ΔHAMD-17 was significantly more improved in participants taking tricyclic antidepressants at 0 - 2 weeks and 0 - 4 weeks.

From our literature assesment, we determined that targeted therapy can undermine the influence of biopsychosocial factors and allow natural resilience to bring depression to an end. Many mental activities is not exclusively individual, but depends on the sociocultural context as people are part of a community.

**Conclusions:**

Depressive disorders can be understood as a rather habitual dysregulation of human behavior which, unlike normal behavior, is not limited by natural resilience in time and severity. Our investigations looked at polymorphisms impacting serotonergic, dopaminergic and adrenergic neurotransmissions and enzymes.

While some associations were found, it did not match our literature findings. For future investigation, epidemiological and pathogenetic biological psychiatric research should be aimed at identifying biopsychosocial factors that frustrate the natural recovery process.

**Disclosure of Interest:**

None Declared

